# Economic Estimation of the Losses Caused by Surface Water Pollution Accidents in China From the Perspective of Water Bodies’ Functions

**DOI:** 10.3390/ijerph13020154

**Published:** 2016-01-22

**Authors:** Hong Yao, Zhen You, Bo Liu

**Affiliations:** School of Geography Science, Nantong University, Nantong 226019, China; yaohong80@126.com (H.Y.); youzhen@ntu.edu.cn (Z.Y.)

**Keywords:** surface water pollution accidents, loss estimation, damage to water bodies’ functions

## Abstract

The number of surface water pollution accidents (abbreviated as SWPAs) has increased substantially in China in recent years. Estimation of economic losses due to SWPAs has been one of the focuses in China and is mentioned many times in the Environmental Protection Law of China promulgated in 2014. From the perspective of water bodies’ functions, pollution accident damages can be divided into eight types: damage to human health, water supply suspension, fishery, recreational functions, biological diversity, environmental property loss, the accident’s origin and other indirect losses. In the valuation of damage to people’s life, the procedure for compensation of traffic accidents in China was used. The functional replacement cost method was used in economic estimation of the losses due to water supply suspension and loss of water’s recreational functions. Damage to biological diversity was estimated by recovery cost analysis and damage to environmental property losses were calculated using pollutant removal costs. As a case study, using the proposed calculation procedure the economic losses caused by the major Songhuajiang River pollution accident that happened in China in 2005 have been estimated at 2263 billion CNY. The estimated economic losses for real accidents can sometimes be influenced by social and political factors, such as data authenticity and accuracy. Besides, one or more aspects in the method might be overestimated, underrated or even ignored. The proposed procedure may be used by decision makers for the economic estimation of losses in SWPAs. Estimates of the economic losses of pollution accidents could help quantify potential costs associated with increased risk sources along lakes/rivers but more importantly, highlight the value of clean water to society as a whole.

## 1. Introduction

Surface water pollution accidents (abbreviated as SWPAs) have become the most dominant type of environmental accident in China [1,2,3,4]. The Songhuajiang River accident on 13 November 2005, the cadmium pollution incident in Beijiang River in December 2005, the water crisis with odorous tap water caused by algae blooms in Wuxi City in Jiangsu in May 2007, have all seriously affected human health, caused large-scale social panic and posed sudden threats to the environmental safety of China [2,3,5,6,7,8].

One problem decision makers usually encounter in accident assessment and risk prevention is that the degrees and costs of surface water accidents are hard to be totally and well understood and identified. The influence of major water pollution accidents is widespread and they can cause damages to multiple aspects, including financial losses, environmental deterioration and negative social influence. Economic losses in surface water accidents can be related to social, ecological, and policy-related factors [9]. When reliable estimates of economic losses from accidents can be set, they can potentially define problems for policy makers and direct focus to areas with the greatest potential economic costs. However, economic loss estimation in accidents is a pretty fuzzy and complicated task. Assigning economic values to an environmental function or ecological service has been widely debated, with researchers employing a variety of methodologies [9,10,11]. However, the systematic and integrated estimation of the damages incurred due to SWPAs has rarely been discussed.

Economic estimation of loss in SWPAs has been one of the focuses in China and has been mentioned many times in the Environmental Protection Law of China promulgated in 2014 [12]. Potential cost is particularly important in both accident evaluation and cost-benefit analysis of policy making and implementation. The effects of the exposure in accidents depend on the physical and chemical nature of the pollutants and on the properties of the biological systems, as well as on the physical environment and its sensitivity. However, the documentation of economic harm from SWPAs in China is limited, and it is nearly impossible to devise an exact method to perform a valuation of the losses caused by SWPAs.

Wetlands (lakes and rivers) play a critical role in the global water cycle, carbon cycle, nitrogen cycle, climate change and ecological development and they provide habitats for wildlife [6,7,8]. They also provide functions such as drinking water, recreation, and aesthetic benefits, all of which can be negatively influenced by water pollution accidents. Pollution related to drinking water protection areas may affect the quality of drinking water and cause tap-water suspension. Water suspension may also occur in irrigation water and industrial production water supplies. Recreational angling and boating activities can be impeded by pollutant-driven taste and odor problems or the inf;uence of toxic substances [13]. Water users are less likely to swim, boat, and fish during accidents and post-accidents due to health risks, unfavorable water appearance, or unpleasant odors [9]. Environmental property values can decrease with the declines in surface water quality, groundwater quality and soil quality along pollution belts. All these negative impacts brought about by SWPAs should be economically assessed.

Thus the aim of this study is to establish a methodology for evaluating of the damage, both direct and indirect, caused by surface water accidents. Economic losses of accidents, classified in various types based on the various aquatic environmental functions mentioned above, were estimated referring to environmental value estimation techniques. At the end of the paper, we apply the proposed estimation method to the Songhuajiang River accident, the most important surface water pollution accident that has occurred in recent years in China.

## 2. Methods

### 2.1. Damage List

**Figure 1:**
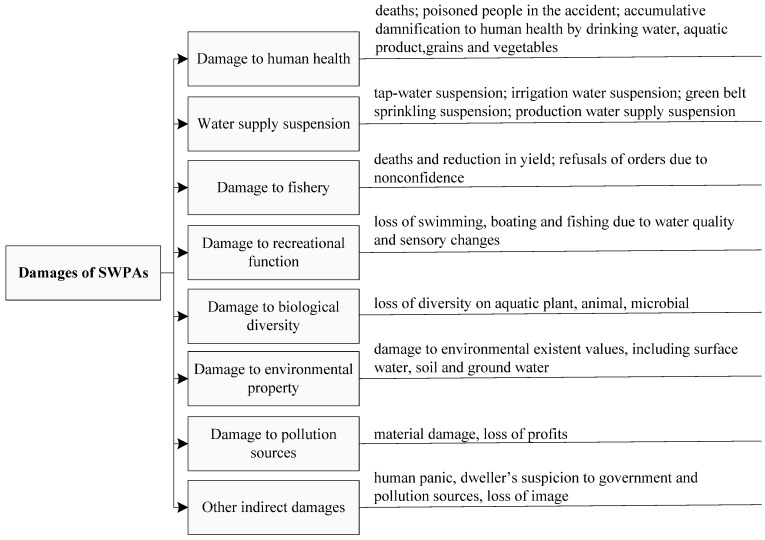
Potential damages caused by SWPAs.

The damages were classified in eight types (Figure 1). The potential damages originated from SWPAs may affect human health, affect the recreational functions of surface water, cause large areas of fish deaths and reduction in aquatic product yields, intermittent water supplies and decrease environmental property values. *etc.* Furthermore, indirect damages, such as public panic, distrust, suspicion of the government, managers and enterprises may occur after an accident. Detailed explanations of these eight components are provided below.

### 2.2. Damage to Human Health

Here human health damage refers to both the loss of human lives and the people poisoned (slightly, severely and very severely) due to a pollution accident. The number of damaged people could be determined during the process of the accident’s remediation. Further some cumulative and persistent substances discharged in SWPAs may bring potential long-term negative effects on human health via environmental media. It is difficult to assess this potential hazard and in this study this damage was seen as a part of damage to environmental property, the valuation method of which is discussed in Section 2.7.

The economic assessment of human health is a rather complex and controversial issue. Nevertheless, it is the only way to establish a cost when a pollution accident has direct consequences on people. The most readily available and simplest procedure to follow in the estimation of human health losses is probably the analytical scheme proposed by Ronza [14]. This method is based on weighing up the economic values which can be attributed to the deaths and poisoned people. Referring to this estimation scheme, the evaluation of economic losses related to human life and health is the sum of the two components expressed in the following equation: the loss of the deaths plus the cost of the diverse categories of poisoned people.

(1) $$L_{HH}\;=V_{d}N_{d}\;+\sum_{k\;=\;1}^{3}V_{k,p}N_{k,p}$$

*L_HH_*: the economic loss of damage to human health (CNY)
*V_d_*: the economic valuation of one fatality (CNY )
*N_d_*: the number of fatalities (-)
*V_k,p_*: the economic valuation of one person poisoned in the category *k* (CNY )
*N_k,p_*: the number of poisoned people in the category *k* (-)
*k* = 1: slightly poisoned people; *k* = 2: severely poisoned people; *k* = 3: very severely poisoned people.

#### 2.2.1. Economic Valuation of a Fatality

Obviously, the problem of attributing an economic value to human life has important moral and social implications. A number of methods have been developed to assess the cost of a fatality [15,16]. Qualitative verbalizations of willing-to-pay (WTP) for improved health and safety controls are commonly used evaluate the value of a life. The empirical results are interpreted theoretically in terms of preference construction processes [16], but in practice this method encounters ethical dilemmas, such as a reluctance to value life and budget constraints. According to “*the year of potential life lost*” proposed by the U.S. Centers for Disease Control and Prevention in 1982, life is valued in proportion to a person’s potential economic production [15]. The cost of saving an extra life (CSX) relates the value of a human life to the investment and expresses the investment made for saving one extra life by involving life expectancy in the calculation [17]. Based on the utility of life, the life quality index (LQI), has also been used [18].

Considering the availability of public information in China, the valuation method used in the study is based on the income *per capita* and the life expectancy, which is a similar measure to the LQI method proposed by Jatin [18]. Besides, the cost of one life should also include the living cost of the dependents. Thus the cost of one fatality will be the function of the age of the victim and his annual income, the number of relatives depending on him, *etc.* Thus the economic valuation of one fatality contains two parts: the victim’s own loss and the cost of the dependents’ living needs (Table 1).

**Table 1:** The details of evaluating one fatal victim (unit: CNY).

Details of Loss Estimation		*a* ≤ 60	60 < *a* < 75	*a* ≥ 75
Victim’s own loss in age *a*		*UI* (or *RI*) × 20	*UI* (or *RI*) × (80 − *a*)	*UI* (or *RI*) × 5
Cost of dependents’ living needs	living expenses of one elder in age *ae*	60 < *ae* < 75	*ae* ≥ 75	
		*UI* (or *RI*) × (80 − *ae*)	*UI* (or *RI*) × 5	
	living expenses of one child in age *ay*	*UI* (or *RI*) × (18 − *ay*)		

The victim’s own loss is standardized according to annual disposable *per capita* income of urban residents (*UI*) or annual net *per capita* income of rural residents (*RI*) of the region where the victim resides. The life expectancy is assumed to be 80 and the same expectancy was used in the road accident compensation standard prescribed in the Road Traffic Safety Law of China [19]. For the sake of fairness, for victims less than 60 years old the fixed number of years used in the valuation is 20 and for victims more than 75 years old, the fixed number is 5 (Table 1). This exception is also consistent with the regulations in the road accident compensation rules [19]. Dependents comprise the children (less than 18 years old) and the elderly (more than 60 years old). In the valuation of the dependents’ living expenses, the *UI* or *RI* value is just the consumption expenditure standard *per capita* and the duration of compensation dependents obtained is the same as in the victim’s own loss estimation.

Two exceptions must be taken into consideration in some cases. If the age of the victim is unknown, it is assumed to belong to the “*less than 60 years old*” category (Table 1). If the information of the dependents is unavailable, it is assumed that the victim has one ten-year-old child and two seventy-year-old elderly relatives.

#### 2.2.2. Economic Valuation of Poisoned People

The economic losses due to poisoned people are evaluated as the function of their age and the severity of poisoning. The degree of poisoning is divided into three degrees: slight, severe and very severe according to the classification proposed by Ronza [14]. The “very severely” poisoned person indicates the ones that utterly lose the ability to work, while severe ones are partly disabled and slight poisoning has no effect on life and work (Table 2).

Poisoned people’s own loss and the living cost of the dependents are included in the loss estimation. The previously mentioned criteria used in death valuation are also applicable in this part, with multiplication by a grade of poisoning coefficient. The coefficients of slight, severe and very severe poisoning are 0.4, 0.7 and 1, respectively [19]. The duration of sick leaves must be also taken into consideration, which can be estimated as the three times the average days of hospitalization (abbreviated as *dh*) in the region. The cost of sick leaves is the product of average daily wage of the region and the duration. In addition, medical fees must be included. If realistic fees are not available, the average hospitalization expense in the region has been used.

**Table 2:** The details of the loss estimation of one poisoned victim(unit: CNY).

Details of Loss Estimation	Slight	Severe	Very Severe
Poisoned people’s own loss and living cost of the dependents	cost in homologous death × 0.4	cost in homologous death × 0.7	cost in homologous death × 1
Loss of sick leaves	average daily wage × *dh* × 3		
Medical fees	average hospitalization expense		

If the distribution of poisoned people is not available, it is advisable to use 1:3:6 ratios between the numbers of very severely, severely and slightly poisoned people. If the ages of the victims are unknown, they are also assumed to belong to the “*less than* *60 years old*” category (see Table 1). If information about dependents is not available, it is also assumed that the victim has one ten-year-old child and two seventy-year-old elderly relatives. If the number of poisoned people is unknown, it can be estimated according to the relation “the number of the poisoned people = 6 × (the number of the deaths)^0.77^” [20].

### 2.3. Losses Due to Water Supply Suspension

Pollutants in surface water accidents might cause water quality, taste and odor problems. This may lead to water supply suspensions, including suspensions of tap-water for domestic use, farmland irrigation, cities’ green belt sprinkling and industrial water. Thus the most direct functions of surface water will be partly lost temporarily. Water supply suspension may cause substitution consumption or industrial production shutdown. Functional replacement cost analysis is a common method in estimating the economic losses brought about by water supply suspensions [21,22]. The value of this special function may be assessed by estimating the cost of the cheapest water replacement with the same effect (Table 3).

**Table 3:** The formulas and nomenclature used in the loss estimation of water supply suspension.

Formulas	Nomenclatures
*L_WS_* = *L_RS_* + *L_FS_* + *L_GS_* + *L_PS_*	*L_WS_*: the economic loss due to water supply suspension (CNY)
	*L_RS_*: the loss due to tap-water suspension for domestic use (CNY)
	*L_FS_*: the loss due to water suspension for irrigation (CNY)
	*L_GS_*: the loss due to water suspension for green belt sprinkling (CNY)
	*L_PS_*: the loss due to water suspension for industrial production (CNY)
*L_RS_* = *P_RS_* × *q*_1_ × *N_r_* × *d*_1_	*P_RS_*: the price of the replacement (CNY/m^3^)
	*q*_1_: the consumption of tap-water *per capita* per day (m^3^/cap./d)
	*N_r_*: the number of urban population with access to tap-water (cap.)
	*d*_1_: the duration of tap-water suspension (d)
*L_FS_* = *P_FS_* × *q*_2_ × *A_f_* × *d*_2_	*P_FS_*: the price of the replacement (CNY/m^3^)
	*q*_2_: the consumption of water in paddy fieldperhectare per day (m^3^/ha./d)
	*A_f_*: the area of paddy field (hectare)
	*d*_2_: the duration of irrigation water suspension (d)
*L_GS_* = *P_GS_* × *q*_3_ × *A_g_* × *d*_3_	*P_GS_*: the price of the replacement (CNY/m^3^)
	*q*_3_: the consumption of greening land per square meter per day (m^3^/m^2^/d)
	*A_g_*: the area of green belt sprinkling (m^2^)
	*d*_3_: the duration of greening water suspension (d)
*L_PS_* = *P_PS_* × *Q_p_* × *d*_3_	*P_PS_*: the price of the replacement (CNY/m^3^)
	*Q_p_*: the quantity of production water demand per day in the affected region (m^3^/d)
	*d*_3_: the duration of production water suspension (d)

#### 2.3.1. Losses Due to Tap-Water Suspension

In China, when tap-water access is suspended, wells are the usual alternative. Therefore the rural area population is not included in the number of the residents with access to the tap-water replacement and only the urban population was involved in this economic evaluation.

Barreled water is the most common substitution during tap-water suspensions. We compared the prices of 24 kinds of barreled water on China’s current market and the cheapest one (0.9 CNY per liter) was selected as the substitution for the tap-water.

Considering that people will consciously save water during a crisis situation, the minimum value, which is expressed in the standard water quantity for a city’s residential use (GB/T 50331-2002) [23] and much less than the actual quantity of residential usage, has been taken as the consumption of barreled water *per capita* per day in the emergency situation.

#### 2.3.2. Losses Caused by Irrigation Water Suspension

The replacement for irrigation water is artificial rainfall [24]. The cheapest cost of this replacement in China was 0.002 CNY per m^3^ [25]. Water consumption for paddy field per hectare per day could be estimated by the average quantity of irrigation demand all around the nation, which can be found in the Chinese Water Resources Bulletin issued annually by the Ministry of Water Resources of China [26].

#### 2.3.3. Losses Caused by Green Belt Sprinkling Suspension

Artificial rainfall is also the most rational replacement for greenbelt sprinkling suspensions. An empirical datum (0.0022 m^3^/m^2^/d), which is obtained from the total sprinkling area in cities divided by the annual sprinkling water consumption over the nation, was deemed as the water demand for greening belt sprinkling in the city [26].

#### 2.3.4. Losses Due to Industrial Water Suspension

The cheapest substitution for industrial production water might be getting water from some other nearby river by pumps and making it usable after simple pretreatment. The average price of this replacement is about 1.3 CNY per m^3^ based on estimating the cost of electricity and pretreatment [26].

### 2.4. Damage to Fisheries

The loss here refers to the direct damage to fisheries caused by surface water pollution, which has been divided into two parts: aquatic species deaths poisoned by pollutants and decreases in yield because of environmental changes and reduced food supplies. The death proportions in accidents and yield reduction after accidents are both difficult to be precisely and directly distinguished, but the recovery time of aquatic products in polluted water can be roughly estimated by the AQUATOX model, an aquatic ecosystem model developed and proposed by USEPA [27]. Assuming that before the quantities of aquatic products recover, fishing is forbidden, thus the economic loss of damage to a fishery can be expressed as:

(2) $$L_{\;f}=\;{AI}_{\;f}\times rt$$

*L_f_*: the loss due to damage to the fishery (CNY)
*AI_f_*: the annually gross income from fisheries in the polluted water (CNY/year)
*rt*: the recovery time of aquatic products (years)

### 2.5. Damage to Recreational Functions

Recreation, including swimming, boating, angling and some other leisure modes (such as walking along the river bank, landscape appreciation *etc.*) is one of the primary functions of surface water. The replacement cost method is used in this section. It is assumed that all the recreational activities are interrupted for the duration of the pollution episode and the losses due to the recreational activities’ interruption can be expressed as the sum of the four parts: losses of swimming, boating, angling and other activities (Table 4).

**Table 4:** The formulas and details in the loss valuation of recreational functions.

Formulas	Nomenclatures
*L_R_* = *L_SM_* + *L_BT_* + *L_AG_* + *L_LM_*	*L_R_*: the loss of damage to recreation (CNY)
	*L_SM_*: the loss of swimming (CNY)
	*L_BT_*: the loss of boating (CNY)
	*L_AG_*: the loss of angling (CNY)
	*L_LM_*: the loss of other leisure means (CNY)
*L_SM_* = *P_SM_* × *N_SM_* × *d*	*P_SM_*: the price of the replacement for swimming per person (CNY/cap./d)
	*N_SM_*: the number of people swimming in the water per day (cap./d)
	*d*: the duration of the pollution episode (d)
*L_BT_* = *P_BT_* × *N_BT_* × *d*	*P_BT_*: the price of the replacement for boating (CNY/cap./d)
	*N_BT_*: the number of people boating in the water per day (cap./d)
	*d*: the duration of the pollution episode (d)
*L_AG_* = *P_AG_* × *N_AG_* × *d*	*P_AG_*: the price of the replacement for angling (CNY/cap./d)
	*N_AG_*: the number of people angling along the river per day (cap./d)
	*d*: the duration of the pollution episode (d)
*L_LM_* = *L_SM_* + *L_BT_* + *L_AG_*	--

Local statistical data can provide the number of people swimming, boating and angling in the water per day. Swimming pools outdoors are chosen as the replacement for swimming in rivers and the lowest price of admission to local outdoors swimming pools is estimated as the replacement price. The substitution for boating is canoeing in tourist spots and angling could be replaced by paid fishing in parks. The prices of the two alternatives can be obtained by way of interviewing tourist spot operators and the replacements’ prices used in the estimation are still the cheapest ones.

The loss of other recreational functions is multiform and can’t be valued directly. One simple way to assess this loss is to estimate it as a ratio of the sum of the losses due to swimming, boating and angling interruption. Referring to the ratio between the direct and indirect accidental losses proposed by Lanoie and Tavenas [28], a 1:1 ratio is assumed (Table 4).

### 2.6. Damage to Biological Diversity

Pollutants might decrease the richness of aquatic macroinvertebrates, fish, and other aquatic primary producers [29,30,31]. The value of biological diversity is difficult to precisely quantify. In China, there are no special funds for the protection of aquatic biology. After surface water accidents, the most general manual intervention for biodiversity recovery undertaken by local governments in China is to release aquatic fingerlings to the water so as to complement and recover the decreased natural biological population, improve and enhance the biological population structure, and restore biological diversity [13].

The recovery cost method (RCM) is used in this study to assess the economic losses from damage to the biological diversity. Recovery measures mainly refer to releasing fingerlings to the water for a period of time. Correspondingly, the recovery cost is the expense for the aquatic fingerlings. Thus the losses of biodiversity can be expressed as:

(3) $$L_{BD}\;=\;C_{rf}\;\times T$$

*L_BD_*: the loss on biodiversity (CNY)
*C_rf_*: the cost of releasing aquatic fingerlings annually after an accident (CNY/year)
*T*: the duration of the biodiversity resumption (year)

Annual costs for releasing aquatic fingerlings after an accident can be obtained from local statistics departments and the duration of this release behavior can be approximately equal to the period of the biodiversity resumption.

### 2.7. Environmental Property Losses

Pollutants released in the accident deteriorate the water quality and decrease the value of the surface water. Pollutants may also settle in the sediments and the groundwater nearby might also suffer negative consequences due to pollutants’ penetration. Environmental property losses here are defined as these damages and denote the impairment of the value of environmental media, including the surface waters, groundwaters and sediments.

Pollutant clearance cost (PCC) analysis has been applied in this section. We use the cheapest price of pollutant removal from environmental media as the loss of environmental property (Table 5).

**Table 5:** The formulas and nomenclatures of the estimation on environmental property losses.

Formulas	Nomenclatures
*L_EP_* = *C_SW_* + *C_GW_* + *C_SO_*	*L_EP_*: the loss of environmental property (CNY)
	*C_SW_*: the cost of pollutants’ removals from the surface water (CNY)
	*C_GW_*: the cost of pollutants’ removals from the groundwater (CNY)
	*C_SO_*: the cost of pollutants’ removals from the sediment (CNY)
*C_SW_* = *P_SW_* × *V_SW_*	*P_SW_*: the price of removing the pollutants from surface water (CNY/m^3^)
	*V_SW_*: the volume of polluted surface water (m^3^)
*C_GW_* = *P_GW_* × *V_GW_*	*P_GW_*: the price of groundwater remediation (CNY/m^3^)
	*V_GW_*: the volume of polluted groundwater (m^3^)
*C_SO_* = *P_SO_* × *A_SO_*	*P_SO_*: the price of sediment remediation (CNY/m^2^)
	*A_SO_*: the area of polluted sediment (m^2^)

As the volume of polluted groundwater is generally not available, a ratio of the volume of polluted surface water, which can be easily reckoned by way of summing the extension of pollutants’ diffusion, has been used to estimate this parameter. The ratio 1:1 is considered to be moderate [28].

The prices of removing pollutants from environmental media can be obtained by asking related environmental consulting companies devoted to environmental remediation. If this way cannot do, the price of groundwater remediation can be regarded as much as 100 times the cost of surface water remediation to convey the message that it is pretty expensive and the real price might be higher. If accurate information for the price of sediment remediation is not available, it could be obtained using an interest rate based on 1600 CNY per m^2^ in 2000 according to reference [32].

### 2.8. Loss of the Accident Origin

This part refers to the real and financial loss of accident origins. For the accidents that originate at manufacturing enterprises, it includes damage to equipment, the cost of lost wages for closed workshops, the loss of profits for discontinued production, the cost of recruitment and training costs for replacement workers, the reduction in product quality following the accident. For the accidents caused by traffic accidents, it may include the damage to vehicles and the loss of carried materials. Based on the survey of accident causes, the loss of this part can be simply summed according to the actual conditions.

### 2.9. Other Indirect Losses

SWPAs could bring some other negative consequences, such as human panic, fishery order reduction due to decreased confidence, residents’ suspicion of governments’ decisions, morale effects on coworkers, administrative costs (of the personnel, the department of health and safety, the prevention initiatives of local administators), the loss of image for government and enterprises, *etc.*

Although these indirect losses evidently exist and have an important impact on society, they are related to personal psychology and perspectives. It is hard to exactly reckon their economic values. The simplest way to estimate these losses is to estimate them as a ratio of the sum of the direct losses, which is the loss of human health, water supply suspension, damage to fisheries and recreation. Lanoie and Tavenas proposed a 1:1 ratio between direct and indirect accidental costs [28]. Rikhardsson and Impgaard gave a similar value (with a 66%contribution of the direct costs) [33]. We therefore suggest a ratio 1:1 with the sum of the loss of human health, water supply suspension, damage to fisheries and recreation.

## 3. Case Study: Economic Loss Estimation for the Songhuajiang River Pollution Accident

**Figure 2:**
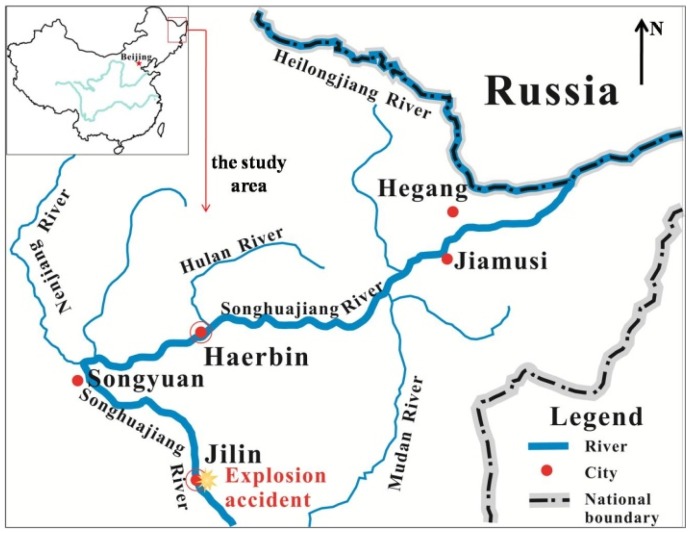
Map of the polluted area in the Songhuajiang River pollution accident.

In 13 November 2005, a major surface water accident happened in China as a result of an explosion at the biphenyl production workshop of Jilin Petrochemical Company, one of branches of Petro China, in Jilin City, Jilin Province, (Figure 2). The accident caused one hundred tones of benzene and related compounds to pour into the Songhuajiang River. The pollutants flowed along the river and formed a 1200-kilometer-long pollution zone crossing two Chinese provinces: Jilin and Heilongjiang (Figure 2). Pollutant concentrations exceeding the thresholds regulated in the environmental quality standards for surface water lasted for 42 days [34]. Water supplies in downstream cities: Songyuan, Haerbin, Hegang, Jiamusi (Figure 2), were all suspended for 3–4 days. Residents affected by the accident were as many as twenty million. Besides, the pollution plume crossed the national border and engendered negative consequences in Russia too. Due to the unavailability of relevant information, the estimation of economic losses in Russia due to the accident were not included in this paper.

The data used in the economic loss estimation was mainly obtained from the 2006 Statistical Yearbook of Jilin Province, the 2006 Statistical Yearbook of Heilongjiang Province and the 2006 statistical yearbooks of the five polluted cities [35,36]. All data have been scaled to their 2005 values. The potential total economic value of the damages caused from this major environmental pollution accident was assessed as 2263 billion CNY using the calculation procedure mentioned above. This value is 2.48 times the sum of the gross domestic products of Jilin Province (362 billion CNY) and Heilongjiang Province (551 billion CNY) in 2005 (Table 6).

The proportions of loss items in the total losses and in the direct losses were both listed in the fourth and fifth column (Table 6). The majority of the total loss corresponds to the damage to environmental property (99.1% of the total loss) (Table 6), followed by the loss of biological diversity (0.44% of the total). All direct losses (environmental property loss, biological diversity loss and other indirect losses were excluded) add up to 5.3 billion CNY (Table 6), far more than the 70 million CNY valuation reported by the government [35,36].

**Table 6:** The details of the economic losses of the Songhuajiang pollution accident.

Items	Specific Items	Economic Loss (Thousand CNY)	Proportion of the Total Loss (%)	Proportion of the Total Direct Loss (%)
Damage to human health		8.6 × 10^3^	3.8 × 10^−4^	0.2
	Deaths	3.2 × 10^3^	1.4 × 10^−4^	0.1
	Poisoned	5.4 × 10^3^	2.4 × 10^−4^	0.1
Damage due to water supply suspension		2.1 × 10^6^	9.2 × 10^−2^	38.8
	Tap-water for residential use	2.1 × 10^6^	9.1 × 10^−2^	38.7
	Irrigation water	333	1.5 × 10^−5^	0.01
	Sprinkling supply	4	1.7 × 10^−7^	0
	Production water	6.9 × 10^3^	3.0 × 10^−4^	0.1
Damage to fisheries		3.2 × 10^6^	0.14	59.6
Damage to water’s recreational functions		1.7 × 10^4^	7.7 × 10^−4^	0.3
	Swimming	854	3.8 × 10^−5^	0.02
	Boating	2.7 × 10^3^	1.2 × 10^−4^	0.05
	Angling	5.1 × 10^3^	2.3 × 10^−4^	0.10
	Other leisure modes	8.7 × 10^3^	3.8 × 10^−4^	0.2
Damage to biological diversity		9.9 × 10^6^	0.4	-
Environmental property loss		2.2 × 10^9^	99.1	-
	Surface water	1.6 × 10^7^	0.7	-
	Groundwater	1.6 × 10^9^	72.5	-
	Sediment	5.8 × 10^8^	25.9	-
Loss of the pollution source of the accident		6.1 × 10^4^	2.7 × 10^−3^	1.1
Other indirect losses		5.3 × 10^6^	0.2	-
The total loss		2.3 × 10^9^	100	
All direct loss		5.3 × 10^6^		100

The details of the economic losses itemized by region were also described (Table 7). Haerbin is the largest city along with the water flow and suffered the largest proportion (43%) of the total loss, followed in order by Jiamusi, Songyuan, Hegang and Jilin. The spatial distribution of the economic loss in the major pollution accident was consistent with the government's data and public perception in the two provinces [35,36].

**Table 7:** The details of the economic loss itemized by region (thousand CNY).

Items	Jilin	Songyuan	Haerbin	Jiamusi	Hegang
Damage to human health	8.6 × 10^3^	0	0	0	0
Damage to water supply suspension	2.9 × 10^3^	1.3 × 10^5^	1.3 × 10^6^	3.5 × 10^5^	2.5 × 10^5^
Damage to fisheries	7.5 × 10^5^	3.6 × 10^5^	1.4 × 10^6^	5.7 × 10^5^	6.7 × 10^4^
Damage to water’s recreational functions	3.7 × 10^3^	2.4 × 10^3^	8.4 × 10^3^	2.1 × 10^3^	942
Damage to biological diversity	2.3 × 10^6^	1.1 × 10^6^	4.5 × 10^6^	1.8 × 10^6^	2.1 × 10^5^
Environmental property loss	1.5 × 10^8^	3.0 × 10^8^	9.7 × 10^8^	5.6 × 10^8^	2.6 × 10^8^
Loss of the pollution source of the accident	6.1 × 10^4^	0	0	0	0
Other indirect losses	8.3 × 10^5^	4.9 × 10^5^	2.8 × 10^6^	9.2 × 10^5^	3.2 × 10^5^
The total loss	1.5 × 10^8^	3.0 × 10^8^	9.8 × 10^8^	5.6 × 10^8^	2.7 × 10^8^
Proportion of the overall area	6.65%	13.42%	43.19%	24.95%	11.79%

For real accidents, the economic assessment of damage could vary greatly. This is due to diverse reasons and among them the most relevant is that the living standards, fishery income and the prices of replacements of the area where the accident occurs do not have the same value. These factors make them little comparable to each other.

The estimates on the economic valuation of human health losses are probably conservative. Only deaths and poisoned persons were included in the damage. In fact, human health might suffer long-term damages through polluted drinking water, aquatic products, grains and vegetables if the pollutants are persistent and have cumulative effects. Because this damage is hard to value alone, this part was not included in the loss of human health.

In the economic estimation of the damage to water’s recreational functions, we assumed that people do not substitute a nearby and unpolluted lake or river for the polluted water while they go to different sites. In fact, most users may have other free alternatives but the data on these substitutions are not available. Thus this assumption might overestimate these losses.

The loss of environmental property in the case afforded the biggest proportion of the total loss in the accident. However this estimation using the expense for environmental media remediation probably still underestimated the real losses because some media, especially the groundwater and the sediment can hardly be repaired due to the lack of mature on-site remediation technologies [37,38,39,40]. The same analysis is also applicable on the valuation of the losses due to damage to biological diversity. This indicates that complete environmental remediation is a very hard thing to accomplish and implies that the prevention of pollution accidents should be the priority in environmental risk management.

## 4. Conclusions

The number of SWPAs has increased substantially in China. Estimates on the economic losses of accidents could help society quantify potential costs associated with increased risk sources along lakes/rivers but more importantly highlight the value of clean water to all society. However, it is nearly impossible to devise an exact method to perform a valuation of the losses caused by SWPAs. The procedure proposed in the paper should be a good approximation.

In the valuation of damages to people’s life, compensation in traffic accidents in China was analyzed as appropriate to be used. The replacement cost method is used in the economic estimation of the damage to water’s recreational functions. Moreover, it has to be noted that the economic lossess estimated for real accidents can sometimes be influenced by social or political factors, such as the authenticity and accuracy of data obtained from governmental statistical departments, and accident origins. Besides, one or more aspects in the method might be overestimated, underrated or even ignored. However, the results of this assessment may be important to help decision makers comprehensively interpret potential costs associated with SWPAs.
